# Turnover of protein phosphorylation evolving under stabilizing selection

**DOI:** 10.3389/fgene.2014.00245

**Published:** 2014-07-23

**Authors:** Christian R. Landry, Luca Freschi, Taraneh Zarin, Alan M. Moses

**Affiliations:** ^1^Département de Biologie, Université LavalQuébec, QC, Canada; ^2^Institut de Biologie Intégrative et des Systèmes (IBIS), Université LavalQuébec, QC, Canada; ^3^Network for Research on Protein Function, Structure, and Engineering (PROTEO), Univeristé LavalQuébec, QC, Canada; ^4^Department of Cell and Systems Biology, University of TorontoToronto, ON, Canada; ^5^Department of Ecology and Evolutionary Biology, University of TorontoToronto, ON, Canada; ^6^Center for Analysis of Genome Evolution and Function, University of TorontoToronto, ON, Canada

**Keywords:** protein phosphorylation, evolutionary turnover, molecular switches, molecular rheostats, protein evolution, molecular evolution, cell signaling

## Abstract

Most proteins are regulated by posttranslational modifications and changes in these modifications contribute to evolutionary changes as well as to human diseases. Phosphorylation of serines, threonines, and tyrosines are the most common modifications identified to date in eukaryotic proteomes. While the mode of action and the function of most phosphorylation sites remain unknown, functional studies have shown that phosphorylation affects protein stability, localization and ability to interact. Two broad modes of action have been described for protein phosphorylation. The first mode corresponds to the canonical and qualitative view whereby single phosphorylation sites act as molecular switches that either turn on or off specific protein functions through direct or allosteric effects. The second mode is more akin to a rheostat than a switch. In this case, a group of phosphorylation sites in a given protein region contributes collectively to the modification of the protein, irrespective of the precise position of individual sites, through an aggregate property. Here we discuss these two types of regulation and examine how they affect the rate and patterns of protein phosphorylation evolution. We describe how the evolution of clusters of phosphorylation sites can be studied under the framework of complex traits evolution and stabilizing selection.

## INTRODUCTION

The rate of discovery of new protein forms is increasing with the growing sensitivity of biochemical, analytical and bioinformatics tools (Smith et al., 2013). We now contemplate the idea that a large fraction of biological diversity originates in mechanisms that regulate protein expression and functions posttranscriptionally and posttranslationally. Among the major sources of posttranslational regulation and cellular complexity are posttranslational modifications (PTMs; Jensen, 2006), which are additions of peptides, chemical groups or other complex molecules to proteins that modify their activity, stability, degradation, localization, and ability to interact ( Sprang et al., 1988; Madeo et al., 1998; Vazquez et al., 2000; Khmelinskii et al., 2009). Hundreds of such modifications have been reported in the literature and some of these appear to be playing dominant roles, at least in terms of occurrence. Protein phosphorylation on serines, threonines, and tyrosines dominates by an order of magnitude the number of experimental PTMs recorded in common databases (Khoury et al., 2011; Lu et al., 2013). This domination likely derives from biases resulting from the long historical interest for protein phosphorylation (Cohen, 1982), from the more advanced state of experimental identification methods for these PTMs and also for biological reasons, for instance because of the large number of protein kinases in eukaryotic genomes that can perform the necessary enzymatic reactions. The impact of protein phosphorylation on the regulation and deregulation of protein functions in human diseases such as Alzheimer’s disease (Grundke-Iqbal et al., 1986; Alonso et al., 1996), and the numerous gains and losses of phosphorylation in cancer cells (Reimand and Bader, 2013; Reimand et al., 2013) suggest that it plays a major role in proteome regulation and in complex cellular phenotypes. For these reasons, there has been much interest in the recent years for understanding how these PTMs evolve (Moses and Landry, 2010). However, the study of phosphorylation site evolution has met several difficulties that derive from the complex mapping between PTMs and protein functions.

## MOLECULAR MECHANISMS OF PROTEIN REGULATION BY PHOSPHORYLATION

Phosphorylation sites play roles in allosteric and orthosteric regulation of proteins (Nussinov et al., 2012). Allosteric regulation acts through long-distances and involves a conformational change of the protein, while orthosteric regulation occurs at the active site of an enzyme or at the interface between a protein and another molecule. The study of protein phosphorylation has been historically centered on the role of individual phosphorylation sites. Indeed, a single phosphorylation site may have dramatic effects in regulating protein functions. For instance, the phosphorylation of tyrosine 527 on the protein kinase and oncogene Src inactivates the protein through the interaction of this modified residue with its SH2 domain, which closes the kinase into an inactive conformation (Frame, 2002). Because single phosphorylation sites in specific cases play key roles in protein regulation, mutations at these sites may have complex organismal phenotypes. For instance, mutation of Ser47 on the Drosophila circadian clock protein PER modifies its interactions with other circadian proteins and lengthens adult locomotor activity from 24 to 31 h (Blau, 2008; Chiu et al., 2008). It has become clear that the simplistic view of one phosphorylation site – one function cannot be generalized to all phosphosites. Proteins are often multi phosphorylated and the different sites may affect each other’s functions (Cohen, 2000).

**FIGURE 1:**
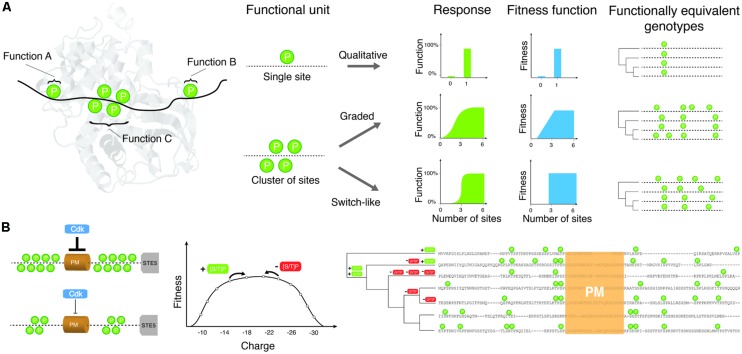
**The relationship between site phosphorylation, localization and protein functions determines how much conservation is expected among species under purifying or stabilizing selection.** **(A)** Toy examples of phosphorylation sites (indicated as “P”s) and cluster of sites and how they may affect protein functions individually or collectively. Phosphorylation sites regulate three putative functions A, B, C. The aggregate function of phosphorylation sites affects the fitness function of the protein and thus determines how many possible equivalent genotypes may give rise to equivalent functions or fitness. Only few possible examples are shown to illustrate the complex relationships expected and their impact on the evolution of phosphorylation profiles and many more are possible. **(B)** Shows a possible fitness landscape for CDK inhibition of Ste5. Ste5 inhibition is proportional to the charge (twice the number of phosphorylated residues) in the disordered region surrounding the PM domain. Evolutionary changes that create CDK consensus sites ([ST]-P) will increase the strength of the inhibition, while changes that destroy consensus sites will reduce the strength of inhibition. The stabilizing selection model suggests that as long as the total strength of inhibition is within an acceptable range, the exact number and location of phosphorylation sites will drift nearly neutrally. A sequence alignment of the disordered regions surrounding the PM domain of Ste5 from *S. cerevisiae* and related yeasts is shown on the right. During evolution consensus sites are gained and lost (+ [ST]-P or - [ST]-P) on the phylogenetic tree, leading to a large diversity in number and location of phosphorylation sites in this region.

Multisite phosphorylation is the premise of more complex types of regulation, for instance concerted regulation and modular regulation (reviewed in Salazar and Hofer, 2007). In the first case, all phosphorylation sites on a protein regulate one or more protein functions in a concerted manner (**Figure 1**). In the second case, groups of phosphorylation sites are organized into modules of multiple sites found in a short distance in a particular domain or disordered region of a protein and each cluster regulates a particular and independent function. Each of the mechanisms described above has its own complexity in terms of effects on the protein and the dynamics of activation, and may thus affect the evolution of these sites (**Figure 1**). It is important to note that these mechanisms are not mutually exclusive. They are typically combined with various types of logic to encode patterns of substrate protein activity. For example, phosphorylation of some sites might “prime” (or increase the probability of phosphorylation) of other sites within the same region through the binding of other proteins that enhance the efficiency of the kinase (Cohen, 2000; Koivomagi et al., 2013; McGrath et al., 2013). In other cases, several sequential regulatory steps have been shown to be required for protein function (Yuan et al., 2002).

Multisite phosphorylation is associated with complex dynamic responses in signaling cascades. The number of sites being phosphorylated and their chronological order of modification can lead to graded or switch-like responses (**Figure 1**), depending on several parameters such as enzyme and substrate concentrations, binding parameters and the kinase/phosphatase processivity (Salazar and Hofer, 2007). One example of graded response involves the enhancement of p53 binding to CREB-binding protein (CBP). The p53 transactivation domain mediates the interaction with CBP and HDM2. Phosphorylation of Thr18 in this domain regulates the qualitative binding (on/off) of HDM2. However, the binding to CBP is regulated in a graded manner, with phosphorylation events contributing additively to the binding energy of p53 to CBP (Lee et al., 2010). Accordingly, it is the sum of the effect that provides an appropriate function to the sites. Another recent example of aggregate effect comes from the circadian rhythm protein FRQ in *Neurospora*, which has more than 100 phosphorylation sites. It was recently demonstrated that the phosphorylation of FRQ is progressive and leads to a buildup of charge in one region of the protein that eventually leads to its degradation (Menet and Rosbash, 2011; Querfurth et al., 2011). A similar mechanism was found to control membrane association of Ste5 in the yeast mating pathway (Serber and Ferrell, 2007). The feature of interest here is that each phosphorylation site does not have a precise role but rather contributes to an aggregate property. The aggregate property was demonstrated experimentally in only few cases but clusters of phosphorylation sites are so abundant in proteomes that a large fraction of proteins could be regulated this way. Large-scale studies have indeed found that phosphorylation sites tend to localize in dense clusters of serines and threonines, which often tend to be phosphorylated by the same kinases (Moses et al., 2007a; Schweiger and Linial, 2010), supporting the hypothesis that a fraction of phosphorylation sites could regulate protein functions in a concerted way rather than acting as individual switches. However, these large-scale datasets need to be interpreted with caution. As it is the case for single phosphorylation sites (see below), clusters of phosphorylation sites could also appear as a result of non-functional phosphorylation. The tendency of phosphorylation sites to cluster could in this case result from the fact that regions accessible to protein kinases are not randomly distributed and form cluster of accessible sites that are susceptible to these phosphorylation events.

## EVOLUTION OF PHOSPHORYLATION SITES

The observation that a large fraction of proteins are posttranslationally modified led to the hypothesis that changes in PTM may contribute to a large fraction of phenotypic variation within species and divergence among species (Moses and Landry, 2010). Many studies have thus examined the conservation and divergence of PTMs, particularly phosphorylation sites. Given the diversity of molecular functions that can be regulated by phosphorylation sites either individually, or when they are found in a multi-site phosphorylated protein, it is expected that their patterns of evolution would also be diverse. For example, in the classical paradigm where a single phosphorylation event leads to a conformation change that alters enzyme activity (Cohen, 1982), one predicts that the phosphorylation site and its position are conserved by purifying selection, as long as the regulation of that enzyme activity is important. Indeed, some well-characterized examples are highly conserved sites over evolution (Rittenhouse et al., 1986; Landry et al., 2009; Tan et al., 2009; Beltrao et al., 2012). However, surprisingly, other well-characterized phosphorylation sites that regulate enzyme activity are not conserved (Hwang and Fletterick, 1986; Landry et al., 2009; Boulais et al., 2010; Nguyen Ba and Moses, 2010; Freschi et al., 2011, 2014; Levy et al., 2012) suggesting that regulation of those enzymes is not critical, or that other mechanisms exist that make phosphorylation sites and their positions unessential. While the phenotypic consequences of the changes in individual sites among species is still mostly unknown – with a few spectacular exceptions (Lynch et al., 2011) – the estimates of the relative rate of phosphorylation site evolution based on large samples led to some challenging conclusions. Some studies concluded that phosphorylation sites are generally under strong evolutionary constraints, i.e., that they evolve much slower than non-modified residues (e.g., Gray and Kumar, 2011), while others estimated that the average constraint imposed on proteins by their phosphorylation is relatively weak (e.g., Landry et al., 2009; studies reviewed in Levy et al., 2012).

Beyond the fact that variation in the different methods and datasets may contribute to some of the disagreements in estimating phosphorylation site conservation, much of the debate comes from the fact that some authors focused their attention on cases where purifying selection is strong whereas others focused on cases where there is no or little purifying selection. On the first hand, in cases where phosphorylation is known to play an important role in regulating the protein, phosphorylation sites are often strongly conserved as predicted (Landry et al., 2009; Nguyen Ba and Moses, 2010; Beltrao et al., 2012). On the other hand, the more challenging observation is that large numbers of uncharacterized sites are poorly conserved among species. There are several possible reasons why sites would evolve quickly. The first is that databases reporting large-scale data on phosphorylation may be populated with a significant fraction of false-positive identifications, i.e., sites that are not actually phosphorylated in cells. Although this limitation contributes little to our understanding of the evolution of phosphorylation sites, it will be an important challenge to be addressed by investigators developing instruments and analytical tools. Another possible scenario is that the rate of evolution is elevated because a significant fraction of sites are species-specific, i.e., they have evolved only recently by directional selection and tests of conservation among species reject the hypothesis that they are under evolutionary constraint. While this hypothesis is of biological interest, there is currently very little data supporting this possibility (but see Jensen et al., 2006; Kim and Hahn, 2011; Lynch et al., 2011), at least not on a scale that would affect significantly the results of analyses performed on thousands of phosphorylation sites.

One other possible mechanism that would explain why many phosphorylation sites evolve as non-modified residues would be that they provide no function to the protein, i.e., they result from non-functional encounters between kinases and their substrates (Lienhard, 2008; Landry et al., 2009). Because kinases are highly processive enzymes and their recognition motifs are highly degenerate (Ubersax and Ferrell, 2007), many collisions between kinases and proteins may result in the inconsequential phosphorylation of residues. This cause is obviously extremely difficult to demonstrate because it is almost impossible to show that some trait or molecular feature has no function, as it is impossible to test all possible parameters that could reveal its role experimentally. However, there are lines of evidence that support this model. For instance, bona fide functional phosphorylation sites are more conserved than the ones for which no evidence is available (Landry et al., 2009; Nguyen Ba and Moses, 2010; Freschi et al., 2014). In addition, the relative rate of phosphorylation site conservation decreases with protein abundance and increases with the stoichiometry of phosphorylation, another observation consistent with a model by which the prevalence of non-functional phosphorylation increases with protein abundance, due to the increased probability of encounters between kinases and other proteins (Levy et al., 2012). However, this observation is also consistent with a higher rate of false positive phosphorylation sites being identified in mass spectrometry studies for high abundance proteins. We note that two of these explanations (false positives, non-functional encounters) posit no or limited biological significance to poorly conserved phosphorylation sites, whereas species specific directional selection would indicate great biological significance for these sites. A final possible explanation for the elevated rate of evolution of phosphorylation site is the rapid turnover of sites caused by the weak constraint on their localization on the protein. This is the case that we consider further here, where rapidly evolving phosphorylation sites do have biological functions, but function is not strongly dependent on each individual site, as discussed above in Section “Molecular Mechanisms of Protein Regulation by Phosphorylation.”

## STABILIZING SELECTION ACTING ON CLUSTERS OF PHOSPHORYLATION SITES

The turnover of phosphorylation sites found in clusters of sites is an appealing hypothesis to explain at least a fraction of rapidly evolving phosphorylation sites (Landry et al., 2009). Indeed, a large fraction of phosphorylation sites are overrepresented in disordered regions of proteins where they occur in clusters of several juxtaposed sites (Moses et al., 2007a; Landry et al., 2009; Schweiger and Linial, 2010) and could act as groups rather than individual sites as described in Section “Molecular Mechanisms of Protein Regulation by Phosphorylation”(**Figure 1**). Sites could be gained and lost within a given region with limited effect on the overall function, but contributing to an increased rate of evolution if one considers sites individually. Different clusters could also play similar roles and diverging species could lose and gain these clusters without any significant change in function (Drury and Diﬄey, 2009). This model, which represents a previously undescribed form of “stabilizing selection” (Burger and Lande, 1994; Charlesworth, 2013a,b), suggests that clusters of sites rather than the sites themselves are the functional unit. This model leads to several predictions that can be tested using existing data: (i) protein functions regulated by phosphorylation should be preserved despite the fact that phosphorylation sites move positions; (ii) phosphorylated residues that occur in clusters should evolve faster because any change in position would be identified as an evolutionary modification; (iii) the number rather than the position of phosphorylation sites within clusters should be preserved over evolution; (iv) sites that are gained and lost between orthologs proteins should be gained and lost within these clusters. While there are no clear experimental data supporting most of these predictions, there are several observations that are consistent with them.

Observations supporting the first prediction come from proteins involved in DNA replication that are regulated by cyclin dependent kinases (CDKs). These proteins show conserved regulation in animals and fungi but diverge in the position and number of clusters of CDK phosphorylation sites, suggesting that functions may be preserved despite changes in phosphorylation patterns (Moses et al., 2007b). Additional evidence comes from the large-scale study of CDK-dependent sites in budding yeasts where it was suggested that phosphorylation sites present in the model species but absent in closely related species could in fact be present in these other species but at other positions (Holt et al., 2009). The second prediction has been tested by (Nguyen Ba and Moses, 2010) who examined whether gains and losses of phosphorylation sites were more permissive in proteins with a high number of phosphorylation sites but no significant evidence was found against the null hypothesis. Evidence supporting predictions (iii) and (iv) comes from the analysis of yeast and mammalian phosphoproteomes. In the first case, it was shown that despite the low rate of phosphorylation site conservation (here the phosphorylation status of the proteins was compared between species or between paralogs), the actual number of phosphorylation sites tended to be maintained over evolution between homologous proteins (Freschi et al., 2011) or group of proteins (Beltrao et al., 2009). Similarly, orthologs of bona fide targets of protein kinases were found to contain conserved clusters of phosphorylation sites, often with little amino acid sequence conservation (Lai et al., 2012). Finally, it was shown that birth and death of phosphorylation sites in proteins tended to be clustered in space (Freschi et al., 2011, 2014), supporting the last prediction of the model.

More work will be needed along these lines to test whether the stabilizing selection model can apply to clusters of phosphorylation sites. Under this model, a phosphorylation cluster would be considered as a quantitative trait where selection does not act on a particular site but rather on the number or density of sites. In this model, each site represents a single locus that contributes to a “complex trait.” Deviation from the trait optimum is associated with a fitness cost that would increase or decrease following a given function (see, e.g., Charlesworth, 2013a) when phosphorylation sites are added or removed by mutations in this region (**Figure 1**). As mutation-selection-drift equilibrium is reached after a long divergence time, there will be a large diversity of “genotypes” that encode the trait. Even if the optimal trait value is the same in all species, if the number of phosphorylation sites (loci) that contribute to the trait is large, a minimal level of conservation will be observed for the actual phosphorylation sites. For example, CDK-mediated inhibition of Ste5 membrane localization is mediated by 8 phosphorylation sites in *S. cerevisiae* (**Figure 1**; Serber and Ferrell, 2007; Strickfaden et al., 2007). The strength of the inhibition is proportional to the net charge of the Ste5 PM domain after phosphorylation by CDKs. In evolutionary terms, the net charge of the PM domain is a quantitative trait whose value simply doubles the sum of the CDK phosphorylation sites in the PM domain. If a mutation occurs at a phosphorylation site, and renders it unphosphorylatable, the net charge of the PM domain will decrease by 2. On the other hand, if a mutation creates a new phosphorylation site (by adding a serine or threonine followed by a proline) in this region, the value of the trait will increase by 2. Assuming that there are several values of the trait that are compatible with functional inhibition by CDK, the PM domain will be free to drift in sequence space through any “genotype” that has enough phosphorylation sites to retain function. At mutation-selection-drift equilibrium, a large number of genotypes will coexist (**Figure 1**), corresponding to different positions and numbers of phosphorylation sites in the PM domain. This stabilizing selection model has been applied to explain the rapid evolution of transcription factor binding sites in highly conserved developmental enhancers in drosophila (Ludwig et al., 2000). In one recent simulation study, clusters of transcription factor binding sites were shown to evolve spontaneously simply because there are a much larger number of genotypes with many binding sites that encode the trait than “simple” genotypes with few binding sites (He et al., 2012). Whether this type of model can explain the origin of phosphorylation site clusters is currently a promising open research question.

## CONCLUSION

Given the current knowledge on the function of clusters of phosphorylation sites, there is a need for the development of new evolutionary models or adaptation of existing ones that take into account the fuzziness of phosphorylation site position and density. Although to our knowledge the stabilizing selection model has not been explicitly applied to the evolution of phosphorylation sites, the distribution of kinase site consensus matches in the absence of selection has been calculated (Lai et al., 2012). This represents a neutral null hypothesis for the evolution of phosphorylation site clusters. Deviations from this model were exploited to predict substrates for several protein kinases because orthologs of bona fide substrates contain more matches to the phosphorylation site consensus than expected in the absence of selection. Knowledge on kinase recognition motifs can thus be exploited to examine the neutral evolution of phosphorylation sites and thus to derive null hypothesis regarding their evolution.

The study of protein phosphorylation will also need more experimental studies on the function of cluster of phosphorylation sites in order to learn the general principles by which their aggregate properties emerge from their combination. This will allow for instance to estimate the relationship between the number of sites, their density and positions, and protein function and organismal fitness. Most evolutionary studies performed so far, with few exceptions (Landry et al., 2009), rely on comparative data among species and not on within species polymorphisms. Detailed analysis of within species variation coupled with functional studies could help estimate the distribution of genotypes under selection-mutation-drift equilibrium for given clusters of sites. Above all, the adaptation of more complex models to the evolution of PTMs will provide a better global picture of the evolutionary forces acting on phosphoproteomes. At the same time, these models will have the potential to contribute to biochemical studies whereby evolutionary observations will guide experimental investigators in studying the code of phosphorylation site regulation.

## AUTHOR CONTRIBUTIONS

Christian R. Landry drafted the manuscript with contributions from Alan M. Moses. Luca Freschi contributed concepts, discussion and to manuscript edition. Taraneh Zarin and Luca Freschi contributed the figure.

## Conflict of Interest Statement

The authors declare that the research was conducted in the absence of any commercial or financial relationships that could be construed as a potential conflict of interest.
